# Moral Distress in Hospitals During the First Wave of the COVID-19 Pandemic: A Web-Based Survey Among 3,293 Healthcare Workers Within the German Network University Medicine

**DOI:** 10.3389/fpsyg.2021.775204

**Published:** 2021-11-18

**Authors:** Juliane Nora Schneider, Nina Hiebel, Milena Kriegsmann-Rabe, Jonas Schmuck, Yesim Erim, Eva Morawa, Lucia Jerg-Bretzke, Petra Beschoner, Christian Albus, Julian Hannemann, Kerstin Weidner, Susann Steudte-Schmiedgen, Lukas Radbruch, Holger Brunsch, Franziska Geiser

**Affiliations:** ^1^Department of Psychosomatic Medicine and Psychotherapy, University Hospital Bonn, Medical Faculty, Bonn, Germany; ^2^Department of Psychosomatic Medicine and Psychotherapy, University Hospital Erlangen, Friedrich-Alexander University Erlangen-Nürnberg (FAU), Erlangen, Germany; ^3^Department of Psychosomatic Medicine and Psychotherapy, Ulm University Medical Center, Ulm, Germany; ^4^Department of Psychosomatics and Psychotherapy, Medical Faculty and University Hospital, University of Cologne, Cologne, Germany; ^5^Department of Psychotherapy and Psychosomatic Medicine, Faculty of Medicine, Dresden University of Technology, Dresden, Germany; ^6^Department of Palliative Medicine, University Hospital Bonn, Medical Faculty, Bonn, Germany

**Keywords:** COVID-19, moral distress, healthcare, healthcare workers, mental health, depression, anxiety

## Abstract

**Objective**: The present study aimed to investigate the correlation between moral distress and mental health symptoms, socio-demographic, occupational, and COVID-19-related variables, and to determine differences in healthcare workers’ (HCW) moral distress during the first wave of the COVID-19 pandemic.

**Method**: Data from 3,293 HCW from a web-based survey conducted between the 20th of April and the 5th of July 2020 were analyzed. We focused on moral distress (Moral Distress Thermometer, MDT), depressive symptoms (Patient Health Questionnaire-2, PHQ-2), anxiety symptoms (Generalized Anxiety Disorder-2, GAD-2), and increased general distress of nurses, physicians, medical-technical assistants (MTA), psychologists/psychotherapists, and pastoral counselors working in German hospitals.

**Results**: The strongest correlations for moral distress were found with depressive symptoms, anxiety symptoms, occupancy rate at current work section, and contact with severe acute respiratory syndrome coronavirus 2 (SARS-CoV-2). Nurses and MTA experienced significantly higher moral distress than physicians, psychologists/psychotherapists, and pastoral counselors. The average level of moral distress reported by nurses from all work areas was similar to levels which before the pandemic were only experienced by nurses in intensive or critical care units.

**Conclusion**: Results indicate that moral distress is a relevant phenomenon among HCW in hospitals during the COVID-19 pandemic, regardless of whether they work at the frontline or not and requires urgent attention.

## Introduction

The worldwide wave of infection with the novel coronavirus (SARS-CoV-2) was classified as a pandemic by the WHO on 12th March 2020 (World Health Organization, 2020). The COVID-19 pandemic poses a major challenge to health systems worldwide, and healthcare workers (HCW) play an essential role in coping with this global crisis (Petzold et al., 2020). When they stop working as a result of acute stress, exhaustion, and mental illness (Petzold et al., 2020), limited functioning of the health care system may result (Shultz et al., 2016). Previous research revealed that the COVID-19 pandemic adversely affects the psychological well-being and mental health of HCW in hospitals (Chen et al., 2020; Morawa et al., 2021). Therefore, it is essential to identify factors causing stress or mental illness to thwart the (temporal) loss of HCW during the ongoing and future pandemics.

An emerging factor in this context is moral distress (Giannetta et al., 2020). It is assumed to occur when a person considers a certain action to be morally right but is prevented from acting according to his or her ethical beliefs by hierarchical or institutional restraints (Jameton, 1984). Past research has shown that failing to tackle HCWs’ moral distress have adverse effects on their personal and professional lives (Allen et al., 2013) and can promote turnover among HCW (Fournier et al., 2007; Sasso et al., 2016). Moral distress might even harm patients due to its potential negative effect on quality and safety of patient care (McCarthy and Gastmans, 2015). This stresses the importance of gaining a deeper understanding of the construct to support HCW.

### Background

Moral distress frequently occurs among HCW in hospitals (Fournier et al., 2007; Brown-Saltzman et al., 2015; McCarthy and Gastmans, 2015). During a pandemic moral distress is even increased and associated with more demanding and strenuous working conditions (Borges et al., 2020; Jacobs and Manfredi, 2020), for instance, severely limited communication with patients and their relatives due to restraining orders and the fear of contagion (Jacobs and Manfredi, 2020). Especially at the beginning of a pandemic, staff is often not thoroughly trained in infection prevention. As such moral distress is positively associated with general distress (Alkrisat, 2016) and decreased well-being (Lamiani et al., 2017). Furthermore, moral distress is correlated with anxiety (Sasso et al., 2016; Jacobs and Manfredi, 2020), and the development of depressive symptoms among HCW (Sasso et al., 2016; Lamiani et al., 2018).

However, so far research on moral distress in the medical sector mostly focused on nurses (Lamiani et al., 2017; Jacobs and Manfredi, 2020). Nurses often complained higher moral distress compared to physicians (Mehlis et al., 2018; Pergert et al., 2019). It has been proposed that one reason why nurses might be particularly at risk for experiencing moral distress is that they have to act more often contrary to their ethical and moral beliefs without possessing definite power of decision (Lomis et al., 2009; Sajjadi et al., 2017). This is also true for other medical professions like psychologists/psychotherapists, pastoral counselors, and medical-technical assistants (MTA; MTA duties include assistance during surgery, taking care of patients before and after surgery, and the measurement of their body functions; Akademie für Gesundheitsberufe, Universitätsklinikum Ulm, 2021). For instance, during the COVID-19 pandemic MTA duties included working in direct patient care. They often worked in intensive care units where they took care of severely ill COVID-19 patients (Vereinigung Medizinisch-Technischer Berufe in der Deutschen Röntgengesellschaft e.V, 2021). Furthermore, novel research on mental burden during the COVID-19 pandemic revealed increased levels of depressive symptoms and mental distress among MTA compared to physicians (Morawa et al., 2021). Such results highlight the importance to close the knowledge gap of the occurrence of moral distress among other medical professions.

Occupational variables like contact with SARS-CoV-2 infected patients or contaminated material at work are potential sources of conflicts on protection management (Jacobs and Manfredi, 2020). Occupancy rate displays another potential source of moral distress because an increased occupancy at the current work section results in less time per patient and more general workload (Jacobs and Manfredi, 2020). During the first wave of the pandemic, some wards were highly used, while others had a reduced occupancy rate. The relationship between professional experience and moral distress is inconsistent (Ohnishi et al., 2010; Hamaideh, 2014).

Regarding the influence of demographic variables, previous research reveals inconsistent results concerning the relationship between gender and moral distress (O’Connell, 2015; Shoorideh et al., 2015). Age also seems to be of importance. Significantly higher levels of moral distress were observed in younger nurses (Hamaideh, 2014; Wolf et al., 2019).

### Objective

We aimed to explore moral distress among HCW working in hospitals during the first wave of the COVID-19 pandemic by answering the following research questions:

I. Do HCW experience significantly higher moral distress in our sample during the COVID-19 pandemic than HCW in comparable studies conducted before the onset of the pandemic?
II. Are increased general distress, depression and anxiety symptoms, gender, age, professional experience, occupancy rate, contact with SARS-CoV-2, and the number of confirmed COVID-19 cases associated with moral distress? Specifically, we predicted a positive relationship between increased general distress, depression and anxiety symptoms, as well as occupancy rate and contact with SARS-CoV-2 with moral distress among HCW. We also expected the increasing numbers of confirmed COVID-19 cases in Germany as an indicator of the (rapid) spread of the pandemic to be positively associated with moral distress among HCW. Age was supposed to be negatively correlated with moral distress. No clear hypothesis for gender and work experience could be formulated.
III. Are there differences in the prevalence of moral distress between various medical professions?

## Materials and Methods

### Background and Data Collection

The background for this study came from a research group on resilience in religion and spirituality funded by the German Research Foundation. Data collection was performed within the so-called VOICE survey as a collaboration of five university hospitals. Data evaluation was performed as part of the collaborative research project egePan Unimed within the newly set-up German Network University Medicine. The aim of egePan Unimed is to examine and coordinate management concepts of the pandemic in Germany and internationally, to evaluate their practicability using scientific methods, and to manage them within a framework plan. Superior aims include an adequate control of resources within a region to avoid an inefficient occupancy and intensive care supply in an inpatient setting and case management for both hospitalized and non-hospitalized patients.

Data collection for the VOICE online survey took place between April 20 and July 5, 2020. The link was provided through mailing lists (employees, professional associations, hospital boards, etc.) and intranet advertising by the psychosomatic departments of the university hospitals of Bonn, Erlangen, Ulm, Cologne, and Dresden. Participation was also encouraged by several other hospitals and online platforms. The 15min survey consisted of 77 items and was conducted in German. It could be accessed *via* two academic online survey tools, Unipark^1^ and SoSci Survey.^2^ Inclusion criteria for participation were a minimum age of 18years, employment as a HCW in Germany, and the submission of informed consent.

### Measures

The 11-point single-item Moral Distress Thermometer (MDT; Wocial and Weaver, 2013) was used to assess moral distress. After been given a brief explanation of the term moral distress (“Moral distress is a form of distress that occurs when you believe you know the ethically correct thing to do, but something or someone restricts your ability to pursue the right course of action.”; Wocial and Weaver, 2013, S.169), participants rated how much moral distress they have been experiencing related to work in the past 2weeks on a scale ranging from 0=“None” to 10=“Worst possible.”

Existing literature was screened for reference values of moral distress measured by the MDT before the onset of the pandemic. Wocial and Weaver (2013) validated the MDT among 529 nurses working in inpatient settings in US hospitals. Further reference values included a US sample of 41 nurses working in general pediatric inpatient units (Rodrigues et al., 2018), 167 US nurses working in critical care units (Wolf et al., 2019), a German sample of 39 physicians and 50 nurses working in a university hospital with advanced cancer inpatients (Mehlis et al., 2018), and 262 German nurses working in intensive care units (Graeb, 2019).

Participants were asked to rate general distress during and before the COVID-19 pandemic (five-point *not at all* – *very strong* response scale). The difference between the items (during the pandemic – before the pandemic) was calculated for further analysis to assess an increase in general distress through the pandemic. A positive difference indicates increased general distress during the COVID-19 pandemic.

Depressive and anxiety symptoms were measured by the four-item Patient Health Questionnaire (PHQ-4; Löwe et al., 2010), which consists of a two-item depression scale (Patient Health Questionnaire-2, PHQ-2) and the two-item Generalized Anxiety Disorder Screener (GAD-2; 4-point *not at all – nearly everyday* response scale). Sum scores for the PHQ-4 and GAD-2 could reach up to 6, with values of three or higher considered clinically significant. The Spearman-Brown coefficient was 0.75 for the PHQ-2 and 0.77 for the GAD-2.

For demographic, occupational, and COVID-19-related variables, participants were asked to state their gender, age, professional experience (“How long have you been involved in patient care?”), the occupancy rate at the current work section (“How occupied has the ward you work in been for the last 2weeks, including today?”), and whether they get in touch with SARS-CoV-2 infected patients or contaminated material at work. Based on data of the Robert Koch-Institute (RKI, Germany), the total number of confirmed COVID-19 cases in Germany at the week of participation (interval: Thursday to Wednesday the following week in each case) was added to the data set.

### Data Analysis

The software IBM SPSS Statistics, Version 27 for Windows was used for statistical analysis. Spearman’s correlation coefficient was used to calculate bivariate relationships between variables (*r*≥0.10=small, *r*≥0.30=medium, and *r*≥0.50=strong correlation coefficient; Cohen, 1988). In the results section, only correlations with relevant effect sizes (*r*≥0.10) are reported. To investigate the complex network of the ultimate association of derived correlates on moral distress, they were included in a multiple regression model with the forced entry method to answer research question two (*β*=≥ 0.10=small, *β*≥0.30=medium, and *β*≥0.50=strong beta coefficients; Cohen, 1988). In the results section, only correlates with relevant effect sizes (*β*≥0.10) are reported. *R^2^* was used as coefficient of determination (*R^2^*≥0.02=small goodness-of-fit, *R^2^*≥0.13=moderate goodness-of-fit, and *R^2^*≥0.26=high goodness-of-fit; Cohen, 1988). Age and professional experience were assessed in groups of different widths and therefore dummy coded before conducting regression analyses. The dummy variables correlating significantly with moral distress were included as predictors in the multiple regression analysis. To answer research question 1 and 3, group differences for moral distress were analyzed by the independent samples *t*-test or Welch’s analysis of variance. Games-Howell *post hoc* analyses for the latter with Bonferroni adjusted *p* values were conducted. Effect sizes (Cohen’s *d*, *η*^2^) are reported for significant results (*d*≥0.20=small, *d*≥0.50=medium, and *d*≥0.80=strong effect; *η*^2^≥0.01=small, *η^2^*≥0.06=medium, and *η^2^*≥0.14=large effect; Cohen, 1988). Values of *p*<0.05 were considered statistically significant except for the case of alpha error correction (then explicitly reported in the text).

## Results

### Participants

The current paper focused on physicians, nurses, MTA (consisting of medical assistants, medical-technical laboratory assistants, medical technical radiology assistants, and pharmaceutical-technical assistants), psychologists/psychotherapists, and pastoral counselors working in German hospitals (*N*=4,001). Participants were excluded if they did not work in patient care (*n*=404), their responses were not available for all variables of interest in the main analyses (*n*=248) or a combination of these criteria (*n*=56). This resulted in a final sample of 3,293 (73% female, 27% male). Table 1 presents characteristics of the total sample and the five medical professions. The sample consisted of 1,149 nurses (35%), 966 physicians (29%), 905 MTA (27.5%), 149 psychologists/psychotherapists (5%), and 124 pastoral counselors (4%). About 872 (27%) were between 31 and 40years old, 825 (25%) between 51 and 60years; 2,486 (76%) indicated working in inpatient care for more than 6years; 2,053 (62%) reported getting in contact with SARS-CoV-2 infected patients or contaminated material at work. Due to the heterogeneous recruitment strategy, the response rate of the total sample could not be measured; however, we can report some response rates for the four university hospitals with the largest proportions of respondents, which were study centers or cooperation partners. The highest average response rate was found for the MTA (16.1%) followed by physicians (9.2%), and nurses (8.0%). The response rate for pastoral workers and psychologist/psychotherapists could not be measured as we had no knowledge about the total number of pastoral workers and psychologist/psychotherapists working at the hospitals at the time of publication. Over 90% of the HCW participated within the first month after the study was launched (April 20–May 19).

**Table 1:** Characteristics of the study sample.

	Total sample	Physicians	Nurses	MTA	Psychologists/psychotherapists	Pastoral counselors
	*N* =3,293	*n* =966	*n* =1,149	*n* =905	*n* =149	*n* =124
**Gender, *n* (%)**						
Female	2,414 (73.3)	567 (58.7)	874 (76.1)	783 (86.5)	122 (81.9)	68 (54.8)
Male	879 (26.7)	399 (41.3)	275 (23.9)	122 (13.5)	27 (18.1)	56 (45.2)
**Age, years, *n* (%)**						
18–30	711 (21.6)	140 (14.5)	322 (28.0)	197 (21.8)	52 (34.9)	0 (0.0)
31–40	872 (26.5)	336 (34.8)	291 (25.3)	194 (21.4)	48 (32.2)	3 (2.4)
41–50	714 (21.7)	230 (23.8)	242 (21.1)	206 (22.8)	21 (14.1)	15 (12.1)
51–60	825 (25.1)	207 (21.4)	248 (21.6)	267 (29.5)	23 (15.4)	80 (64.5)
above 60	171 (5.2)	53 (5.5)	46 (4.0)	41 (4.5)	5 (3.4)	26 (21.0)
**Workplace, *n* (%)**						
University hospital	2052 (62.3)	568 (58.8)	893 (77.7)	448 (49.5)	114 (76.5)	29 (23.4)
Other hospital	1,241 (37.7)	398 (41.2)	256 (22.3)	457 (50.5)	35 (23.5)	95 (76.6)
**Professional experience, *n* (%)**						
<3years	366 (11.1)	125 (12.9)	78 (6.8)	85 (9.4)	54 (36.2)	24 (19.4)
3–6years	441 (13.4)	128 (13.3)	160 (13.9)	98 (10.8)	36 (24.2)	19 (15.3)
>6years	2,486 (75.5)	713 (73.8)	911 (79.3)	722 (79.8)	59 (39.6)	81 (65.3)
**Occupancy rate, *n* (%)**						
Well below average	486 (14.8)	209 (21.6)	121 (10.5)	112 (12.4)	29 (19.5)	15 (12.1)
Slightly below average	1,010 (30.7)	343 (35.5)	277 (24.1)	291 (32.2)	48 (32.2)	51 (41.2)
Average	991 (30.1)	227 (23.5)	414 (36.0)	262 (29.0)	51 (34.2)	37 (29.8)
Slightly above average	530 (16.1)	118 (12.2)	227 (19.8)	151 (16.7)	18 (12.1)	16 (12.9)
Well above average	276 (8.4)	69 (7.1)	110 (9.6)	89 (9.8)	3 (2.0)	5 (4.0)
**Contact with SARS-CoV-2, *n* (%)**						
							Yes	2053 (62.3)	587 (60.8)	680 (59.2)	707 (78.1)	15 (10.1)	64 (51.6)
No	1,240 (37.7)	379 (39.2)	469 (40.8)	198 (21.9)	134 (89.9)	60 (48.4)

*MTA, medical-technical assistant; occupancy rate, occupancy rate at current work section*.

The mean moral distress score in the present sample was 4.08 (*SD*=2.70: range 0–10). The prevalence of depression and anxiety (cut-off-value of ≥3) in the current sample was 21% and 20%. The mean increase in general distress during the COVID-19 pandemic was 0.51 (*SD*=1.26: range −4–4).

### Moral Distress Before and During the COVID-19 Pandemic

Results of independent samples *t*-tests between moral distress of the study sample and scores of reference samples are shown in Table 2. Nurses’ moral distress in our sample (*M*=4.52, *SD*=2.66: range 0–10) was significantly higher than moral distress scores of nurses before the COVID-19 pandemic in inpatient settings in US hospitals (Wocial and Weaver, 2013; *M*=2.9, *SD*=2.5), US nurses in general pediatric inpatient units (Rodrigues et al., 2018; *M*=2.64, *SD*=2.00), and nurses in a German university hospital with advanced cancer inpatients (Mehlis et al., 2018; *M*=2.3, *SD*=2.3: range 0–9), *t*(1,676)=11.81, *p*<0.001, *d*=0.62; *t*(1,188)=4.48, *p*<0.001, *d*=0.71; and *t*(1,197)=5.81, *p*<0.001, *d*=0.84. The effect sizes correspond to medium and strong effects (Cohen, 1988). There was no statistically significant difference between moral distress of nurses across all work sections during the pandemic in the study and US nurses working in critical care units (Wolf et al., 2019; *M*=4.2, *SD*=2.6) as well as German nurses working in intensive care units (Graeb, 2019; *M*=4.46, *SD*=2.57) before the pandemic, *t*(1,314)=1.47, *p*=0.14; *t*(1,409)=0.34, *p*=0.73.

**Table 2:** Results of independent samples *t*-tests between moral distress scores in study sample and reference samples.

Samples	Nurses		*t*	*p*	Cohen’s *d*	Physicians		*t*	*p*	Cohen’s *d*
	*M (SD)*	*n*				*M (SD)*	*n*			
Study sample (VOICE, 2020)	4.52 (2.66)	1,149				3.42 (2.61)	966			
Wocial and Weaver, 2013	2.9 (2.5)	529	11.81	< 0.001	0.62					
Rodrigues et al., 2018	2.64 (2.00)	41	4.48	< 0.001	0.71					
Wolf et al., 2019	4.2 (2.6)	167	1.47	0.143	0.12					
Mehlis et al., 2018	2.3 (2.3)	50	5.81	< 0.001	0.84	1.5 (1.4)	39	4.56	< 0.001	0.74
Graeb, 2019	4.46 (2.57)	262	0.34	0.731	0.02					

*M, mean, S, standard deviation, and p, significance p value. Results are displayed for comparison of each sample with the study sample VOICE (2020). Wocial and Weaver (2013): nurses in US inpatient settings. Rodrigues et al. (2018): nurses in US general pediatric inpatient settings. Wolf et al. (2019): nurses in US critical care units. Mehlis et al. (2018): nurses and physicians in German advanced cancer inpatient units. Graeb (2019): nurses in German intensive care units*.

Moral distress was significantly higher for physicians in our sample (*M*=3.42, *SD*=2.61: range 0–10) compared to physicians working in a German university hospital with advanced cancer inpatients before the COVID-19 pandemic (Mehlis et al., 2018; *M*=1.5, *SD*=1.4: range 0–6), *t*(1,003)=4.56, *p*<0.001, *d*=0.74. The effect size was found to correspond the convention of Cohen (1988) for a medium effect.

### Correlates of Moral Distress

Bivariate correlations between study variables are shown in Table 3. Higher moral distress was significantly (*r*=0.14–0.35, all *p*<0.01) associated with (in descending order of effect size): depressive symptoms, anxiety symptoms, higher occupancy rate at the current work section, having more general distress than before the pandemic, and being in direct contact with SARS-CoV-2 patients or material.

**Table 3:** Spearman correlations for study variables.

Variable	1	2	3	4	5	6	7	8	9
1. Moral distress									
2. Increase in general distress	0.160^**^								
3. GAD-2	0.330^**^	0.231^**^							
4. PHQ-2	0.348^**^	0.186^**^	0.600^**^						
5. Gender	−0.049^**^	−0.063^**^	−0.088^**^	−0.054^**^					
6. Age	−0.060^**^	0.067^**^	0.000	−0.079^**^	0.054^**^				
7. Professional experience	0.034	0.085^**^	0.007	−0.025	0.000	0.556^**^			
8. Occupancy rate	0.222^**^	0.170^**^	0.108^**^	0.126^**^	−0.045^**^	−0.059^**^	−0.008		
9. Contact with SARS-CoV-2	0.137^**^	0.035^*^	0.061^**^	0.040^*^	0.027	−0.045^**^	0.032	0.064^**^	
10. COVID-19 cases	0.062^**^	−0.072^**^	−0.026	−0.009	−0.035^*^	−0.006	0.023	0.150^**^	−0.053^**^

* *p<0.05*.

** *p<0.01, two-tailed*.

*N=3,293. Increase in general distress=difference of general distress before the onset of the COVID-19 pandemic and during the COVID-19 pandemic. A positive value indicated an increase in general distress during the COVID-19 pandemic. For gender, 0=female, 1=male. For age, 1=18–30, 2=31–40, 3=41–50, 4=51–60, and 5=above 60. For professional experience, 1=less than 3years, 0=more than 3years. For occupancy rate, 1=well below average, 2=slightly below average, 3=average, 4=slightly above average, and 5=well above average. For contact with SARS-CoV-2, 0=no, 1=yes. COVID-19 cases=total number of confirmed COVID-19 cases in Germany at the week of participation (interval: Thursday to Wednesday the following week in each case)*.

We performed a multiple linear regression analysis for the total sample to examine the association of derived correlated on moral distress. The model explained 21% (*R*^2^=0.21, adjusted *R*^2^=0.20) of the variance of moral distress in HCW, indicating a moderate goodness-of-fit (Cohen, 1988). Correlates significantly predicted moral distress, *F*(10, 3,282)=84.79, *p*<0.001, *η^2^*=0.21. Table 4 shows regression results using moral distress as the criterion. Higher moral distress was significantly predicted by: depression symptoms, *β*=0.20, *p*<0.001, anxiety symptoms, *β*=0.18, *p*<0.001, occupancy rate, *β*=0.15, *p*<0.001, and being in direct contact with SARS-CoV-2 patients or material, *β*=0.11, *p*<0.001.

**Table 4:** Summary of multiple regression analysis for the total sample (*N*=3,293) using moral distress as the criterion.

Predictor	b 95% CI [LL, UL]	SE	Beta	*t*	*p*
(Intercept)	0.16 [−0.67, 0.98]	0.42		0.38	0.707
Increase in general distress	0.12 [0.06, 0.19]	0.04	0.06	3.53	<0.001
GAD-2	0.32 [0.25, 0.39]	0.04	0.18	8.63	<0.001
PHQ-2	0.38 [0.30, 0.45]	0.04	0.20	9.90	<0.001
Gender	−0.08 [−0.27, 0.11]	0.10	−0.01	−0.81	0.419
Age 18–30	0.45 [0.22, 0.68]	0.12	0.07	3.86	<0.001
Age>60	−0.13 [−0.51, 0.24]	0.19	−0.01	−0.70	0.487
Professional experience	−0.71 [−1.01, −0.42]	0.15	−0.08	−4.69	<0.001
Occupancy rate	0.36 [0.29, 0.44]	0.04	0.15	9.48	<0.001
Contact with SARS-CoV-2	0.59 [0.42, 0.76]	0.09	0.11	6.72	<0.001
COVID-19 cases	9.26E-6 [0.00, 0.00]	0.00	0.06	3.47	<0.001

*b, unstandardized regression weights, CI, confidence interval, LL, lower limit of a confidence interval, UL, upper limit of a confidence interval, SE, standard error, and Beta, standardized regression weights. p, significance p value. Increase in general distress=difference between general distress before the onset of the COVID-19 pandemic and during the COVID-19 pandemic. A positive value indicated an increase in general distress during the COVID-19 pandemic. For gender, 0=female, 1=male. For age 18–30, 1=18–30, and 0=above 30. For age>60, 1=above 60, and 0=18–60. For professional experience, 1=less than 3years, 0=more than 3years. Occupancy rate=occupancy rate at current work section. For occupancy rate, 1=well below average, 2=slightly below average, 3=average, 4=slightly above average, and 5=well above average. For contact with SARS-CoV-2, 0=no, 1=yes. COVID-19 cases=total number of confirmed COVID-19 cases in Germany at the week of participation (interval: Thursday to Wednesday the following week in each case)*.

### Differences in Moral Distress Between Medical Professions

Differences in moral distress were examined for nurses (*M*=4.52, *SD*=2.66: range 0–10), MTA (*M*=4.49, *SD*=2.68: range 0–10), physicians (*M*=3.42, *SD*=2.61: range 0–10), psychologists/psychotherapists (*M*=3.42, *SD*=2.66: range 0–9), and pastoral counselors (*M*=3.08, *SD*=2.36: range 0–9). Experienced moral distress differed statistically significant between medical professions, Welch’s *F*(4, 532.38)=36.22, *p*<0.001, *η^2^*=0.04. The effect size was found to correspond the convention of Cohen (1988) for a small effect. Games-Howell *post hoc* analysis revealed significantly higher moral distress among nurses and MTA compared to the remaining professions (*p*’s<0.001). Results of pairwise comparisons with Bonferroni adjusted alpha error correction are shown in Table 5.

**Table 5:** Games-Howell post-hoc test for pairwise comparisons of moral distress between medical professions.

(I) Profession	(J) Profession	Mean difference I – J, (*SE*)	95% CI for Difference [LL, UL]
1. Physicians	2	−1.10^***^ (0.12)	[−1.55, −0.65]
(*n*=966)	3	−1.07^***^ (0.12)	[−1.54, −0.59]
	4	0.00 (0.23)	[−0.93, 0.92]
	5	0.34 (0.23)	[−0.57, 1.24]
2. Nurses	1	1.10^***^ (0.12)	[0.65, 1.55]
(*n*=1,149)	3	0.04 (0.12)	[−0.43, 0.50]
	4	1.10^***^ (0.23)	[0.18, 2.02]
	5	1.44^***^ (0.23)	[0.54, 2.34]
3. MTA	1	1.07^***^ (0.12)	[0.59, 1.54]
(*n*=905)	2	−0.04 (0.12)	[−0.50, 0.43]
	4	1.06^***^ (0.24)	[0.13, 2.00]
	5	1.41^***^ (0.23)	[0.49, 2.32]
4. Psychologists/psychotherapists	1	0.00 (0.23)	[−0.92, 0.93]
	2	−1.10^***^ (0.23)	[−2.02, −0.18]
(*n*=149)	3	−1.06^***^ (0.24)	[−2.00, −0.13]
	5	0.34 (0.30)	[−0.85, 1.54]
5. Pastoral counselors	1	−0.34 (0.23)	[−1.24, 0.57]
	2	−1.44^***^ (0.23)	[−2.34, −0.54]
(*n*=124)	3	−1.41^***^ (0.23)	[−2.32, −0.49]
	4	−0.34 (0.30)	[−1.54, 0.85]

*** *p<0.0025 according to Bonferroni adjusted alpha error correction*.

*N=3,293. SE, standard error, CI, confidence interval, LL, lower limit of a confidence interval, and UL, upper limit of a confidence interval. MTA, medical-technical assistant*.

## Discussion

This paper provides an account of HCW’s moral distress during the first wave of infection of the COVID-19 pandemic in 2020 by exploring its prevalence and differences among several medical professions working in German hospitals and investigating the relationship between moral distress and mental health symptoms, several socio-demographic, occupational, and COVID-19-related variables.

### Moral Distress Before and During the COVID-19 Pandemic

Significantly higher levels of moral distress among nurses and physicians in our sample compared to the pre-pandemic reference samples revealed that moral distress occurs more frequently during the pandemic than before. A potential cause might be the exposure to a higher number of moral dilemmas (Borges et al., 2020; Jacobs and Manfredi, 2020) or conflicts (Kühlmeyer et al., 2020) associated with the fight against the pandemic. Although nurses in our sample worked in various hospital sections, their moral distress levels were comparable only to those reported before the pandemic among nurses in intensive and critical care units (Graeb, 2019; Wolf et al., 2019). We assume that, in non-pandemic times, moral dilemmas largely concern existential decisions in patient care typical for critical care units (O’Connell, 2015; Jacobs and Manfredi, 2020). In the COVID-19 pandemic, however, these dilemmas may spill over to all care units in the hospital, and additionally, new dilemmas such as the distribution of protective material (Jacobs and Manfredi, 2020), may arise, and might be associated with the observed increase in the general level of moral distress.

### Correlates of Moral Distress

The strongest relationships with moral distress were found in depression and anxiety symptoms, occupancy rate at the current work section, and contact with SARS-CoV-2 patients or contaminated material. This is consistent with previous research linking moral distress to anxiety (Sasso et al., 2016; Jacobs and Manfredi, 2020) and the development of depressive symptoms among HCW (Sasso et al., 2016; Lamiani et al., 2018). However, no causal direction of these associations can be drawn from the data collected. Higher moral distress may lead to increased levels of depression and anxiety, but it is also conceivable that those who are more depressed and/or anxious may be less able to adapt and may experience some situations as more conflictual and morally distressing. Results furthermore confirmed our assumption of a positive relationship between contact with SARS-CoV-2 infected patients or contaminated material and occupancy rate with moral distress. One source of moral distress specific to the COVID-19-pandemic could be caused by situations in which HCW feel that more protection against infection is needed but are not able to provide it for themselves or their patients. An increased occupancy rate at the current work section, which results in less time per patient and more general workload (Jacobs and Manfredi, 2020), was shown to be another potential source of moral distress in the current sample. However, beta coefficients of all mentioned variables were lower than 0.30, implying a small predictive power of moral distress by the individual variables.

Our findings point out that additional research investigating moral distress among HCW in hospitals during a pandemic is crucial to identify organizational as well as individual protective and risk factors linked to its occurrence.

### Differences in Moral Distress Between Medical Professions

Results revealed that moral distress is a phenomenon often experienced by HCW during the COVID-19 pandemic and does not only affect nurses, but also MTA, physicians, psychologists/psychotherapists, and pastoral counselors. Nurses and MTA experienced the highest levels of moral distress in the current sample. For nurses, this is in line with findings of other studies (Mehlis et al., 2018; Pergert et al., 2019) where they reported higher moral distress than physicians. However, MTA’s moral distress levels were equally high as the ones of nurses. Just like nurses, MTA also often may not have the opportunity to act according to their ethical and moral beliefs as they do not possess high degrees of decision-making power, which represents an increased risk of experiencing moral distress (Lomis et al., 2009; Sajjadi et al., 2017). It is possible that this does not apply for pastoral counselors and psychologists/psychotherapists, who, like physicians, often might have more authority and/or autonomy in their field of work than nurses and MTA. Therefore, a promising approach of reducing moral distress among nurses and MTA might be to give them more authority or autonomy in their field of work.

To our knowledge, the present study was the first one to examine moral distress among MTA, psychologists/psychotherapists, and pastoral counselors working in German hospitals. Beyond that, to our knowledge, this is the first study to also compare moral distress among nurses, physicians, MTA, psychologists/psychotherapists, and pastoral counselors in the hospital setting. Even though the moral distress levels of physicians, psychologists/psychotherapists, and pastoral counselors were slightly lower than the ones of nurses and MTA, it can be assumed that they all were affected by the negative correlates of the phenomenon which include: turnover (Sasso et al., 2016; Lamiani et al., 2017), decreased wellbeing (Lamiani et al., 2017), and burnout (Ajoudani et al., 2019; Jacobs and Manfredi, 2020).

### Limitations, Implications, and Recommendations for Future Research

The present study is limited by some points: moral distress was only assessed with one item, which makes it unlikely that we could capture the construct in its entirety. However, this makes the MDT suited for large surveys, and its validity has been shown to be sufficient (Wocial and Weaver, 2013). Another limitation includes the MDT’s non-specific definition of moral distress, leaving the question of the specific situations in which it has occurred unanswered. The wording of the MDT might have had differing effects on the different medical professions and a variant meaning or understanding of the construct could have influenced results. Another limitation lies in the unbalanced sample sizes of subgroups (e.g., medical professions, age groups, and years of professional experience), and a sole examination of HCW in the in-patient healthcare sector. As this limits generalizability of results, they must be interpreted with caution. Furthermore, general distress before the pandemic was assessed retrospectively. The occurrence of a recall bias cannot be excluded here.

Our findings underline that moral distress among HCW needs urgent attention. It is crucial to understand its root causes to support HCW’s mental health, thwart the (temporal) loss of health professionals (Sasso et al., 2016; Lamiani et al., 2017), and improve patient security during the ongoing and future pandemics. Providing HCW of all professions with additional education about moral distress and its consequences (Heilmann et al., 2021) and setting a special focus on their experiences during the COVID-19-pandemic might be a promising starting point to decrease their moral distress (Toronto and LaRocco, 2019) and strengthen their coping capacities (Jacobs and Manfredi, 2020).

Future research in the domain should additionally identify situations, as well as risk and protective factors that lead to moral distress among different medical professions. A follow-up of our survey is in its final phase, and further assessments are planned with the aim of a longitudinal exploration. The gained knowledge could help to develop preventive evidence-based interventions targeted at the causes of moral distress in dealing with the ongoing and future pandemics.

## Conclusion

Workers in health-related professions play an essential role in coping with the COVID-19 pandemic (Petzold et al., 2020). However, up to date, it remains unclear how to maintain their mental health in this exceptional situation (Chen et al., 2020). Results of the present study demonstrated that moral distress is a relevant phenomenon among HCW in hospitals during the COVID-19 pandemic, regardless of whether they work at the frontline or not and requires urgent attention. As the pandemic is likely to have physical and psychological long-term effects on the population (Borges et al., 2020), we can expect that the extreme demands on our health systems and HCW will continue to exist (Frawley et al., 2020). Reducing the occurrence of moral distress and enabling staff to better cope and develop moral resilience is crucial.

## Data Availability Statement

The datasets presented in this article are not readily available because the authors cannot disclose individual level data due to ethical and legal data protection restrictions and have included group level data in the paper and tables. The dataset from this study is held securely in coded form at the Clinic for Psychosomatic Medicine (CPM) as part of the University Clinic and Medical Faculty of Bonn. While national data protection laws and ethical regulations as well as cooperation agreements prohibit the CPM from making the dataset publicly available, requests for access to anonymized or pooled data may be sent to the Data Access Committee of the CPM. Requests to access the datasets should be directed to Sabine Müller, sabine.mueller@ukbonn.de.

## Ethics Statement

The studies involving human participants were reviewed and approved by the Ethics Committee of the Rheinische Friedrich Wilhelm University of Bonn and the Friedrich-Alexander University Erlangen-Nürnberg. The patients/participants provided their written informed consent to participate in this study.

## Author Contributions

YE, EM, FG, PB, and LJ-B designed the study. NH, YE, EM, MK-R, FG, PB, LJ-B, CA, HB, SS-S, and KW conducted the study. JNS analyzed the data and wrote this manuscript. NH and FG contributed to manuscript writing. MK-R, LJ-B, PB, CA, KW, SS-S, LR, JH, and JS provided critical revision of the manuscript for important intellectual content. All authors contributed to the article and approved the submitted version.

## Funding

This study was designed and set up within the research group “Resilience in Religion and Spirituality,” speaker Cornelia Richter, funded by the Deutsche Forschungsgemeinschaft (DFG, German Research Foundation; grant number 348851031). Data management and evaluation were performed in the collaborative research project egePan Unimed funded by the German Federal Ministry of Education and Research (BMBF) as part of the Network University Medicine (NUM) (funding code 01KX2021); and led by Michael Albrecht, Jürgen Graf, Jochen Schmitt, and Michael von Wagner. The funders had no role in the study design; in the collection, analysis, and interpretation of data; in the writing of the manuscript; and in the decision to submit the paper for publication. The corresponding author had full access to all the data in the study and had final responsibility for the decision to submit for publication.

## Conflict of Interest

The authors declare that the research was conducted in the absence of any commercial or financial relationships that could be construed as a potential conflict of interest.

## Publisher’s Note

All claims expressed in this article are solely those of the authors and do not necessarily represent those of their affiliated organizations, or those of the publisher, the editors and the reviewers. Any product that may be evaluated in this article, or claim that may be made by its manufacturer, is not guaranteed or endorsed by the publisher.

## Footnotes

^1^ www.unipark.com

^2^ www.soscisurvey.de

## References

[ref1] Ajoudani F. Baghaei R. Lotfi M. (2019). Moral distress and burnout in Iranian nurses: The mediating effect of workplace bullying. Nurs. Ethics 26, 1834–1847. doi: 10.1177/0969733018779210, PMID: 29938574

[ref2] Akademie für Gesundheitsberufe, Universitätsklinikum Ulm (2021). Operationstechnische Assistenz. Available at: https://www.akademie.uniklinik-ulm.de/ausbildung/ota/ota-beruf.html (Accessed September 3, 2021).

[ref3] Alkrisat M. (2016). Predict moral distress using workplace stress, stress of conscience mediated by coping using Roy adaptation model: A path analysis. J. Nurs. Meas. 24, 477–492. doi: 10.1891/1061-3749.24.3.477, PMID: 28714452

[ref4] Allen R. Judkins-Cohn T. deVelasco R. Forges E. Lee R. Clark L.. (2013). Moral distress among healthcare professionals at a health system. JONAS Healthc. Law Ethics Regul. 15, 111–118. doi: 10.1097/NHL.0b013e3182a1bf33, PMID: 23963112

[ref5] Borges L. M. Barnes S. M. Farnsworth J. K. Drescher K. D. Walser R. D. (2020). A contextual behavioral approach for responding to moral dilemmas in the age of COVID-19. J. Contextual Behav. Sci. 17, 95–101. doi: 10.1016/j.jcbs.2020.06.006, PMID: 32834968 PMC7334906

[ref6] Brown-Saltzman K. Alyssa Fine M. S. Patricia Jakel M. N. (2015). A culture of avoidance: voices from inside ethically difficult clinical situations. Clin. J. Oncol. Nurs. 19, 159–165. doi: 10.1188/15.CJON.19-02AP, PMID: 25840381

[ref7] Chen Q. Liang M. Li Y. Guo J. Fei D. Wang L.. (2020). Mental health care for medical staff in China during the COVID-19 outbreak. Lancet Psychiatry 7, e15–e16. doi: 10.1016/S2215-0366(20)30078-X, PMID: 32085839 PMC7129426

[ref8] Cohen J. (1988). Statistical Power Analysis for the Behavioral Sciences. New York, NY: Academic, 54.

[ref9] Fournier B. Kipp W. Mill J. Walusimbi M. (2007). Nursing care of AIDS patients in Uganda. J. Transcult. Nurs. 18, 257–264. doi: 10.1177/1043659607301301, PMID: 17607063

[ref10] Frawley T. Van Gelderen F. Somanadhan S. Coveney K. Phelan A. Lynam-Loane P.. (2020). The impact of COVID-19 on health systems, mental health and the potential for nursing. Ir. J. Psychol. Med. 38, 220–226. doi: 10.1017/ipm.2020.105, PMID: 32933594 PMC7596574

[ref11] Giannetta N. Villa G. Pennestrì F. Sala R. Mordacci R. Manara D. F. (2020). Instruments to assess moral distress among healthcare workers: A systematic review of measurement properties. Int. J. Nurs. Stud. 111:103767. doi: 10.1016/j.ijnurstu.2020.103767, PMID: 32956930

[ref12] Graeb F. (2019). Ethische Konflikte und Moral Distress Auf Intensivstationen. Wiesbaden: Springer Fachmedien Wiesbaden.

[ref13] Hamaideh S. H. (2014). Moral distress and its correlates among mental health nurses in Jordan. Int. J. Ment. Health Nurs. 23, 33–41. doi: 10.1111/inm.12000, PMID: 23320816

[ref14] Heilmann K. Hinrichs R. Herke M. Richter M. Rathmann K. (2021). Die Bedeutung der “Big Five”-Persönlichkeitsmerkmale für die subjektive Gesundheit und Lebenszufriedenheit im Jugendalter: Ergebnisse des Nationalen Bildungspanels (NEPS). Das Gesundheitswesen 83, 8–16. doi: 10.1055/a-1068-2280, PMID: 31923923

[ref15] Jacobs B. Manfredi R. A. (2020). Moral distress During COVID-19: residents in training are at high risk. AEM Educ. Train. 4:447. doi: 10.1002/aet2.10488, PMID: 33150293 PMC7592830

[ref16] Jameton A.. (1984). Nursing practice: The ethical issues. Available at: http://hdl.handle.net/10822/800986 (May 20, 2021).

[ref17] Kühlmeyer K. Kuhn E. Knochel K. Hildesheim H. Witt V. D. Friedrich O.. (2020). Moralischer Stress bei Medizinstudierenden und ärztlichen Berufseinsteigenden: Forschungsdesiderate im Rahmen der COVID-19-Pandemie. Bundesgesundheitsbl. Gesundheitsforsch. Gesundheitsschutz 63, 1483–1490. doi: 10.1007/s00103-020-03244-2, PMID: 33180160 PMC7659897

[ref18] Lamiani G. Borghi L. Argentero P. (2017). When healthcare professionals cannot do the right thing: A systematic review of moral distress and its correlates. J. Health Psychol. 22, 51–67. doi: 10.1177/1359105315595120, PMID: 26220460

[ref19] Lamiani G. Dordoni P. Argentero P. (2018). Value congruence and depressive symptoms among critical care clinicians: The mediating role of moral distress. Stress. Health 34, 135–142. doi: 10.1002/smi.2769, PMID: 28664611

[ref20] Lomis K. D. Carpenter R. O. Miller B. M. (2009). Moral distress in the third year of medical school; a descriptive review of student case reflections. Am. J. Surg. 197, 107–112. doi: 10.1016/j.amjsurg.2008.07.048, PMID: 19101252

[ref21] Löwe B. Wahl I. Rose M. Spitzer C. Glaesmer H. Wingenfeld K.. (2010). A 4-item measure of depression and anxiety: validation and standardization of the patient health Questionnaire-4 (PHQ-4) in the general population. J. Affect. Disord. 122, 86–95. doi: 10.1016/j.jad.2009.06.019, PMID: 19616305

[ref22] McCarthy J. Gastmans C. (2015). Moral distress: a review of the argument-based nursing ethics literature. Nurs. Ethics 22, 131–152. doi: 10.1177/0969733014557139, PMID: 25505098

[ref23] Mehlis K. Bierwirth E. Laryionava K. Mumm F. H. Hiddemann W. Heußner P.. (2018). High prevalence of moral distress reported by oncologists and oncology nurses in end-of-life decision making. Psycho-Oncology 27, 2733–2739. doi: 10.1002/pon.4868, PMID: 30156350

[ref25] Morawa E. Schug C. Geiser F. Beschoner P. Jerg-Bretzke L. Albus C.. (2021). Psychosocial burden and working conditions during the COVID-19 pandemic in Germany: The VOICE survey among 3678 health care workers in hospitals. J. Psychosom. Res. 144:110415. doi: 10.1016/j.jpsychores.2021.110415, PMID: 33743398 PMC7944879

[ref26] O’Connell C. B. (2015). Gender and the experience of moral distress in critical care nurses. Nurs. Ethics 22, 32–42. doi: 10.1177/0969733013513216, PMID: 24482261

[ref27] Ohnishi K. Ohgushi Y. Nakano M. Fujii H. Tanaka H. Kitaoka K.. (2010). Moral distress experienced by psychiatric nurses in Japan. Nurs. Ethics 17, 726–740. doi: 10.1177/0969733010379178, PMID: 21097971

[ref28] Pergert P. Bartholdson C. Blomgren K. af Sandeberg M. (2019). Moral distress in paediatric oncology: contributing factors and group differences. Nurs. Ethics 26, 2351–2363. doi: 10.1177/0969733018809806, PMID: 30411660

[ref29] Petzold M. B. Plag J. Ströhle A. (2020). Umgang mit psychischer Belastung bei Gesundheitsfachkräften im Rahmen der COVID-19-Pandemie. Nervenarzt 91, 417–421. doi: 10.1007/s00115-020-00905-0, PMID: 32221635 PMC7100457

[ref30] Rodrigues N. P. Cohen L. L. Swartout K. M. Trotochaud K. Murray E. (2018). Burnout in nurses working with youth with chronic pain: A mixed-methods analysis. J. Pediatr. Psychol. 43, 369–381. doi: 10.1093/jpepsy/jsx105, PMID: 29048476

[ref31] Sajjadi S. Norena M. Wong H. Dodek P. (2017). Moral distress and burnout in internal medicine residents. Can. Med. Educ. J. 8, e36–e43. doi: 10.36834/cmej.36639, PMID: 28344714 PMC5344066

[ref32] Sasso L. Bagnasco A. Bianchi M. Bressan V. Carnevale F. (2016). Moral distress in undergraduate nursing students: A systematic review. Nurs. Ethics 23, 523–534. doi: 10.1177/0969733015574926, PMID: 25904547

[ref33] Shoorideh F. A. Ashktorab T. Yaghmaei F. Alavi Majd H. (2015). Relationship between ICU nurses’ moral distress with burnout and anticipated turnover. Nurs. Ethics 22, 64–76. doi: 10.1177/0969733014534874, PMID: 24948793

[ref34] Shultz J. M. Cooper J. L. Baingana F. Oquendo M. A. Espinel Z. Althouse B. M.. (2016). The role of fear-related behaviors in the 2013–2016 West Africa Ebola virus disease outbreak. Curr. Psychiatry Rep. 18:104. doi: 10.1007/s11920-016-0741-y, PMID: 27739026 PMC5241909

[ref35] Toronto C. E. LaRocco S. A. (2019). Family perception of and experience with family presence during cardiopulmonary resuscitation: an integrative review. J. Clin. Nurs. 28, 32–46. doi: 10.1111/jocn.14649, PMID: 30129259

[ref36] Vereinigung Medizinisch-Technischer Berufe in der Deutschen Röntgengesellschaft e.V (2021). Anerkennung für MTA im Kampf gegen COVID-19. Available at: https://www.vmtb.de/de-DE/6292/anerkennung-fuer-mta-im-kundf-gegen-covid-19/ (Accessed September 3, 2021).

[ref37] Wocial L. D. Weaver M. T. (2013). Development and psychometric testing of a new tool for detecting moral distress: the moral distress thermometer. J. Adv. Nurs. 69, 167–174. doi: 10.1111/j.1365-2648.2012.06036.x, PMID: 22607094

[ref38] Wolf A. T. White K. R. Epstein E. G. Enfield K. B. (2019). Palliative care and moral distress: an institutional survey of critical care nurses. Crit. Care Nurse 39, 38–49. doi: 10.4037/ccn2019645, PMID: 31575593

[ref39] World Health Organization (2020). Report No: 73. Coronavirus disease 2019 (COVID-19): situation report. World Health Organization. Available at: https://apps.who.int/iris/handle/10665/331686 (Accessed December 10, 2020).

